# Flunarizine is more effective than topiramate in patients with chronic migraine and medication overuse headache

**DOI:** 10.1186/1129-2377-1-S14-P202

**Published:** 2013-02-21

**Authors:** M Gracia-Naya, N Hernando-Quintana, MJ García-Gomara, S Sánchez-Valiente, C Ríos, S Santos-Lasaosa, JA Mauri, J Artal-Roy, AM Latorre-Jimenez

**Affiliations:** 1 Hospital Miguel Servet, Spain; 2 Hospital Royo Villanova, Spain; 3 Hospital C. Barbastro, Spain; 4 Hospital Clínico Zaragoza, Spain; 5 Hospital San Jorge. Huesca, Spain

## Introduction

Medication overuse headache (MOH) implies secondary headache on a daily or near daily basis, for 15 days or more a month for 3 month and chronic migraine CM is the most common subtypes of MOH in speciality care [1]. Flunarizine and topiramate are considered as first-choice drugs in prophylactic treatment of episodic or transformed migraine^(2)^. Topiramate is considered as first-choice drug in treatment of CM ^(3)^.

## Objetives

We analyze two independent case-series with (CM) and (MOH) according to ICHD-II criteria treated with topiramate or flunarizine as first intention and compare the results.

## Methods

Patients were medication over users and naïve to (oral) prophylactic therapy. In both groups, the main effectiveness variables (reduction in the number of seizures and days with headache at four months of treatment and responder rates) were analysed.

## Results

The study included 348 patients: 188 with flunarizine (88.2% females; mean age: 43.8 ± 13.6) and 160 with topiramate (88.0% females; mean age: 40.2 ± 13.2). No significant differences were found between groups as regards mean age, sex and number of migraines and days with headache in the previous month. There was a significant decrease (0.0001) in the mean number of crisis in the fourth month of treatment, but with no significant difference between them: topiramate (9.3 ± 7.1 to 4.6 ± 6.1) and flunarizine (9.9 ± 7.3 to 4.1 ± 5.0). Mean of days with headaches at four month of treatment, topiramate 9.7±8.5, flunarizine 6.9±8.4 (.0106); the respondent rate was: topiramate 57.9%, flunarizine 72.6% ( .0391). The mean reduction in the number of days with headaches: topiramate 48.2%, flunarizine 63.3% (.0040).

## Conclusions

Both drugs showed effectiveness when used as the preferred drug in the preventive treatment of (CM) and (HOM). Flunarizine offered better results on reduction in the number of days with headaches and minor side effects than topiramate.
